# Effects of Leading Edge Defect on the Aerodynamic and Flow Characteristics of an S809 Airfoil

**DOI:** 10.1371/journal.pone.0163443

**Published:** 2016-09-22

**Authors:** Yan Wang, Xiaojing Zheng, Ruifeng Hu, Ping Wang

**Affiliations:** 1 Key Laboratory of Mechanics on Environment and Disaster in Western China, Ministry of Education, Lanzhou University, Lanzhou, China; 2 School of Energy & Power Engineering, Lanzhou University of Technology, Lanzhou, China; 3 School of Mechano-Electronic Engineering, Xidian University, Xi’an, China; Coastal Carolina University, UNITED STATES

## Abstract

**Background:**

Unexpected performance degradation occurs in wind turbine blades due to leading edge defect when suffering from continuous impacts with rain drops, hails, insects, or solid particles during its operation life. To assess this issue, this paper numerically investigates the steady and dynamic stall characteristics of an S809 airfoil with various leading edge defects. More leading edge defect sizes and much closer to practical parameters are investigated in the paper.

**Methodology:**

Numerical computation is conducted using the SST *k*-*ω* turbulence model, and the method has been validated by comparison with existed published data. In order to ensure the calculation convergence, the residuals for the continuity equation are set to be less than 10^−7^ and 10^−6^ in steady state and dynamic stall cases. The simulations are conducted with the software ANSYS Fluent 13.0.

**Results:**

It is found that the characteristics of aerodynamic coefficients and flow fields are sensitive to leading edge defect both in steady and dynamic conditions. For airfoils with the defect thickness of 6%*t*_c_, leading edge defect has a relative small influence on the aerodynamics of S809 airfoil. For other investigated defect thicknesses, leading edge defect has much greater influence on the flow field structures, pressure coefficients and aerodynamic characteristics of airfoil at relative small defect lengths. For example, the lift coefficients decrease and drag coefficients increase sharply after the appearance of leading edge defect. However, the aerodynamic characteristics could reach a constant value when the defect length is large enough. The flow field, pressure coefficient distribution and aerodynamic coefficients do not change a lot when the defect lengths reach to 0.5%*c*,1%*c*, 2%*c* and 3%*c* with defect thicknesses of 6%*t*_c_, 12%*t*_c_,18%*t*_c_ and 25%*t*_c_, respectively. In addition, the results also show that the critical defect length/thickness ratio is 0.5, beyond which the aerodynamic characteristics nearly remain unchanged. In dynamic stall, leading edge defect imposes a greater influence on the aerodynamic characteristics of airfoil than steady conditions. By increasing in defect length, it is found that the separated area becomes more intense and moves forward along the suction surface.

**Conclusions:**

Leading edge defect has significant influence on the aerodynamic and flow characteristics of the airfoil, which will reach a stable status with enough large defect size. The leading edge separation bubble, circulation in the defect cavity and intense tailing edge vortex are the main features of flow around defective airfoils.

## Introduction

Over the last several decades, there have been great efforts paid to investigate how surface roughness affects the performance of wind turbine. Surface roughness in the wind turbine community is a broad term containing different sizes and shapes of roughness, including the almost non-detectable roughness such as dust or slight erosions up to large-scale roughness like some severe erosion defects or ice accretion geometries[1]. Based on the location of the wind turbines, the blades suffer from various environmental factors that create different blade surface roughness or even defects, forming various airfoil profiles. In recent years, the effects of leading edge erosion or defect on the performance of wind turbine, has jumped into the sight of researchers and engineers and act as a significant negative factor for large scale wind turbines in some challenging environments[2–7], such as offshore regions with severe salt corrosion, desert areas with abrasive sand particles, etc. Though the roles leading edge defect playing in the degradation of wind turbine performance is well known and some initial studies has been conducted, few work has been done to quantify the effects of erosion or defect on wind turbine performance. Previous studies mainly focused on the slight erosion of turbine blade with pits or gouges on the surface[8], or studies with simplified erosion patterns with small amount of variations[3, 9]. Additionally, a majority of these investigations have been conducted through experimental measurements, while the local flow field near the erosion or defect can hardly be presented to demonstrate the influence of leading edge erosion or defect. Therefore, there are still significant gaps in the knowledge of the aerodynamic and flow characteristics for eroded or damaged wind turbine blades.

After several years’ service, wind turbine blades may degrade as a result of erosion or impact damage on the surfaces. The occurrence of leading edge defect can be found only after two years of turbine operation[5], the main reason of which could be the exposure to various forms of precipitation and abrasive airborne particles[7, 10, 11]. Though blade protection has been applied during manufacturing process, quality issues, materials, environmental factors, and other problems also play important roles in the rate of leading edge degenerating[2, 6]. Small air pockets along the leading edge of blade are routinely overlooked and covered up with coating, which expand and contract and wear the surface of wind turbine from the interior of the coating as the blade bends and flexes[2]. Meanwhile, external impingement causes deterioration on the leading edge from the exterior. If repairing is not done or done incorrectly, the chordwise and spanwise cracks may occur and develop, and the health of the blade will be jeopardized. Leading edge erosion or defect has been regarded as an important issue and great challenge for manufacturers and operators in wind power industry[6]. More than 60% energy of a wind turbine is extracted from the outboard section of 0.7*R*-1.0*R* of a blade, which is always operated at a speed of 80–100 ms^-1^ making leading edge defect a commonplace for modern large wind turbines[11], where *R* is the radius of wind turbine.

This study numerically investigates steady and unsteady viscous flow over a stationary airfoil with leading edge defect in the form of cavity. It has been documented that leading edge erosion or defect induce an Annual Energy Production decrease of about 20% for the 1.5MW wind turbine over a period of 5 years operation in an unprotected case[5]. Even though the detrimental effects of the blade erosion or defect are well known qualitatively, their quantitative influences have not been thoroughly revealed until recent years. Sapre & Sareen[8, 10] carried out wind tunnel experiments to study the effects of leading edge erosion on aerodynamic performance of DU 96-W-180 airfoil. Gharali & Johnson[3] simulated the lift coefficient hysteresis loop of an S809 airfoil at three severe erosion conditions, and the eroded airfoil was artificially modeled at the leading edge by a rectangular cavity. Other investigations mainly focused on the effects of ice, dust and insect debris accretion on wind turbine blade performance [12–19].

In this work, the steady and dynamic aerodynamic characteristics as well as flow field contours of detective airfoils are investigated. The computation results of the normal airfoil without defect are compared to experimental results and other simulations for the validation of numerical method. The effects of various defect cavity lengths and thicknesses on the aerodynamic coefficients and flow structures of airfoil are studied comprehensively. According to Gharali & Johnson[3], lift reduction are presented at two erosion lengths of 4%*c* and 14%*c* under dynamic conditions, where *c* is the chord length of the airfoil, and erosion length is the eroded size along the chordwise from leading edge. In addition, study on the erosion effects is one small section of that paper, not much data are presented to demonstrate the aerodynamics of various erosion shapes, nor the elaborate statement on why this phenomenon produce. However, a thorough understanding of aerodynamic characteristics for eroded blade at various erosion extents is conductive to the management and maintenance of wind turbine blades. In present study, much larger defect parameter range is selected, e.g. defect lengths ranging from 0.5%*c* to 14%*c*, defect thicknesses ranging from 6%*t*_*c*_ to 25%*t*_*c*_, and try to determine the critical ratio length/thickness of defect on the aerodynamic performance, where *t*_*c*_ is the thickness of airfoil. Through which we believe that more complete knowledge of the influence of leading edge defect on the flow and aerodynamic characteristics of airfoil can be obtained.

## Physical Model

**Fig 1:**
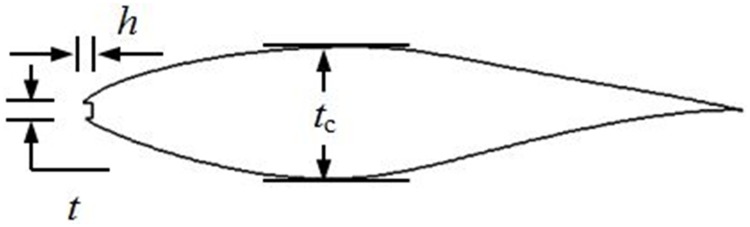
The leading edge defect model for S809 airfoil.

Although actual wind turbine blade is three-dimensional, the profile of leading edge defect is almost the same in spanwise over long time operation, therefore the defect could be considered to be modeled in two-dimension for simplicity. Therefore, two-dimensional (2D) defective airfoil geometry is adopted in present work, which is set as the form of cavity and is firstly proposed by Gharali & Johnson[3]. The S809 airfoil, coming from NREL S family[20], is widely used in wind-turbine design[20]. The airfoil has been shown to be insensitive to surface roughness[21] and can improve the aerodynamics of blade at low and medium wind speeds[22]. Additionally, the airfoil with relative thickness of 20.95% is always used in the outboard section of the blade for megawatt-scale wind turbine[21]. The defect is represented by a rectangular cavity at the leading edge of airfoil, which is quantitatively characterized by the defect length and thickness. Fig 1 presents a schematic illustration of the defective airfoil model. Both steady and dynamic pitching of the airfoil is investigated. In steady state cases, 9 defect lengths and 4 defect thicknesses are considered, i.e., *h*/*c* = 0.5%, 1%, 2%, 3%, 4%, 6%, 8%, 10% and 14%, *t*/*t*_c_ = 6%, 12%, 18% and 25%, where *h* is the defect length, and *t* is the defect thickness. For the defect thickness of *t*/*t*_c_ = 6%, a defect length of 3%*c* is added for a clear understanding of small leading edge defect influence. In dynamic cases, 4 defect lengths are selected based on results of steady simulations at the defect thickness of 12%*t*_c_, i.e. *h*/*c* = 0.5%, 1%, 2%, 3%. The final aim of this study is to promote further understanding of the aerodynamic and flow characteristics of airfoil with growing leading edge defect.

## Computational Methods

The flow field around the airfoil is obtained by numerically solving the two-dimensional Reynolds-Averaged Navier—Stokes equations. The *k*-*ω* SST model, realizable *k*-*ε* model and one-equation S-A model are three popular turbulent models in engineering applications [3, 13, 17]. The computational results of normal airfoil without defect obtained with the above mentioned 3 models are compared to the available experimental data[23] to determine which one to be chosen in present study.

### Computational domain and mesh

**Fig 2:**
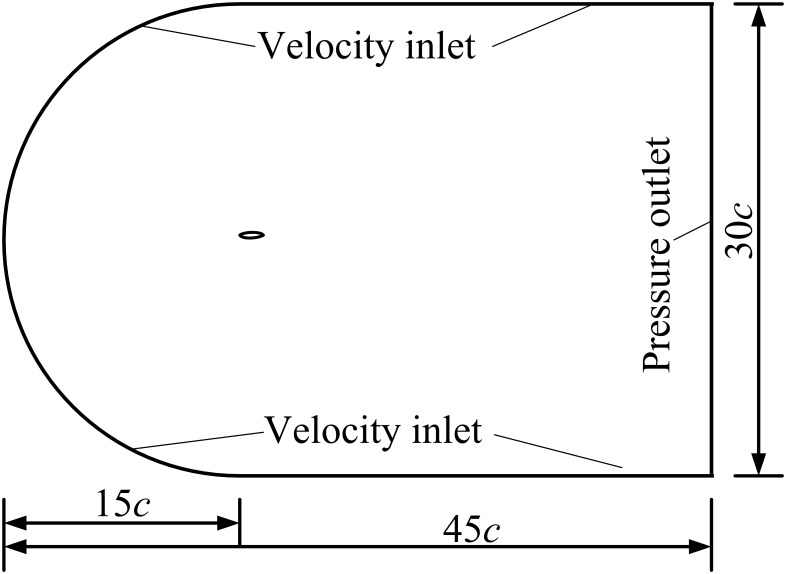
Computational domain.

The domain size is an important factor in computation, and simulation experiments have been conducted to determine proper location of the external boundary to eliminate the far-field boundary influence. It is found that a distance of 15*c* from boundary to airfoil is enough. Therefore, the present computational domain is composed of a semi-circle boundary with radius of 15*c* and a square with length of 30*c*. The airfoil is located at the center of the semi-circle, as shown in Fig 2.

**Fig 3:**
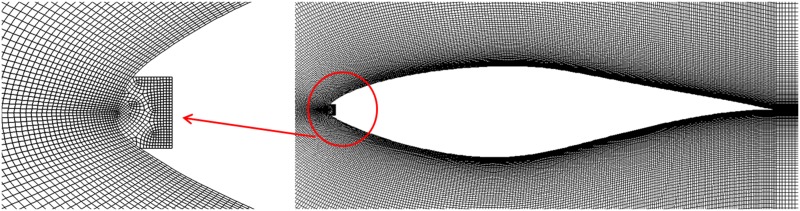
Local enlarged grids for S809 airfoil and the eroded leading edge.

A typical C-type quadrilateral mesh, shown in Fig 3, is adopted in the paper. The total grid element number is about 161,000. Around the airfoil, 500 nodes are distributed with high resolution at the leading and trailing edges. In the wake of the airfoil, 220 nodes and 170 nodes are placed in the horizontal and vertical directions respectively. The height of first element adjacent to the airfoil surface is about 2×10^™5^m, corresponding to *y*^+^≤1 approximately. The local enlarged illustration for the mesh is shown in Fig 3.

### Simulation setup

In steady state case, the simulation is conducted with the angles of attack 4.1°, 8°and 12.2° at the Reynolds number of 10^6^. For dynamic stall cases, unsteady inflow condition with varying velocity direction and magnitude is adopted, and the inflow can be expressed in a sinusoidal pattern:

$$\alpha =\alpha_{mean}+\alpha_{amp}\text{sin}(\omega t)$$ (1)

where *α*_mean_ is the mean angle of attack of the relative velocity, and is 8° in present study, *α*_amp_ is the pitching oscillation amplitude and is set to 10°, *f* is the oscillation frequency which is given as 0.61 corresponding to a reduced frequency of *k* = *ωc*/2*U*_∞_ = 0.026, and *ω* = 2*πf*. The freestream velocity *U*_∞_ is 31.96 m/s which corresponds to a Reynolds number of *Re*_c_ = 10^6^. In the simulation, the airfoil is aligned with the horizontal axis. The parameters adopted here are agree with experiment of Ramsay et al.[23], and the computational results for the smooth airfoil will be compared to the experimental data for validation.

**Fig 4:**
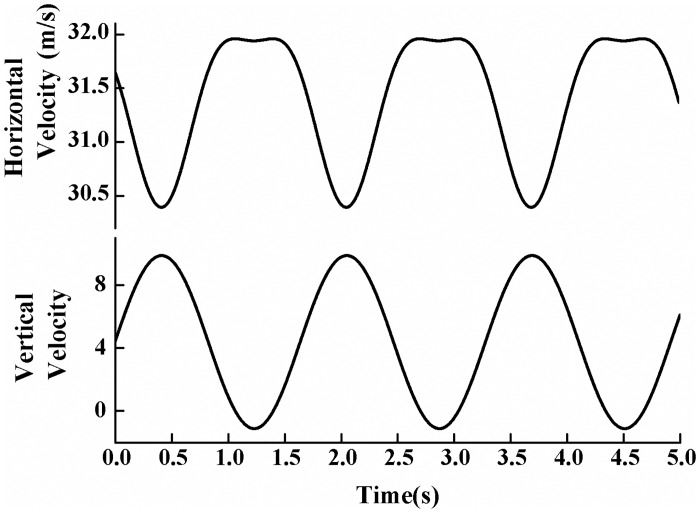
Sinusoidal inlet velocity of the computational domain.

The commercial software ANSYS Fluent 13.0 is used in the computation[24]. Segregated solver and implicit and absolute velocity are adopted to set up the numerical simulation. The SIMPLE algorithm is applied for velocity-pressure decoupling. Second-order upwind scheme is used for spatial discretization. The velocity inlet and pressure outlet boundary conditions are employed in both steady and dynamic cases. In dynamic case, the unsteady inflow condition is realized with the help of the user-defined function (UDF) in ANSYS Fluent 13.0[24]. The function is programmed based on Eq (1) for angle of attack which enters into the decomposition of horizontal and vertical velocity components at each time step, as shown in Fig 4. The no-slip boundary condition is applied at the airfoil surface.

Different time steps have been tested and a time step of 0.003s is found to be sufficiently small to obtain convergent results of lift coefficient *C*_*l*_ and drag coefficient *C*_*d*_.

## Results and Discussion

### Steady state case

#### Numerical validation

**Fig 5:**
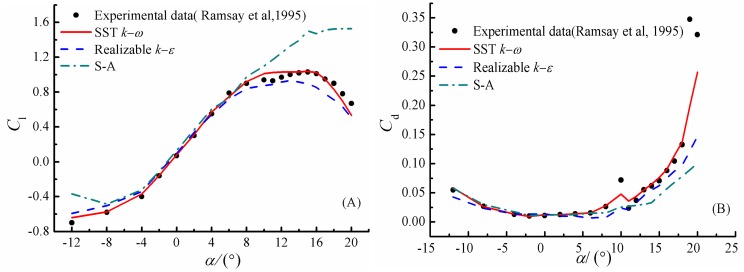
Lift and drag coefficients versus *α* for a smooth airfoil in steady state case. (A) lift coefficient. (B) drag coefficient.

Firstly, the three turbulence models mentioned above, namely the *k*-*ω* SST, realizable *k*-*ε* and S-A model are assessed through comparison with experimental results[23]. In Fig 5, computed lift and drag coefficients of flow past a smooth airfoil are compared with the experimental data of Ramsay et al[23]. It is obviously seen that the SST *k*-*ω* model yields the smallest discrepancy among the three models. For example, it is able to capture the peak lift at *α* = 15° and match the experimental curve well for negative angles of attack. Meanwhile, the predicted drag coefficients by the *k*-*ω* SST model also agree very well with the experimental data. The realizable *k*-*ε* model a little underestimates the lift coefficient at high angles of attack. However, the S-A model prediction of lift coefficient deviate significantly from experiment data when the angles of attack are higher than 10°.

**Fig 6:**
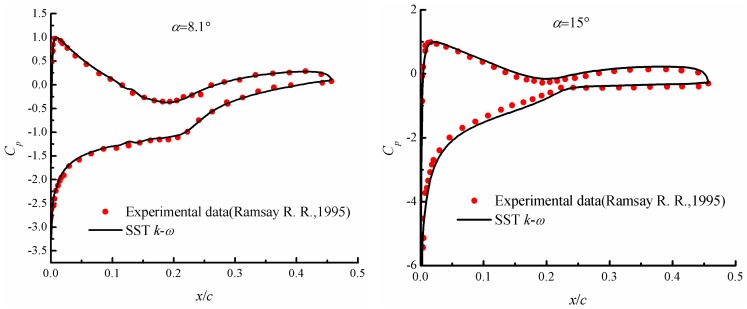
Pressure coefficient computed by *k*-*ω* SST turbulence model.

In order to further evaluate the turbulence model, the pressure coefficients computed by the *k*-*ω* SST model are shown in Fig 6 at the angles of attack 8.1° and 15°. Excellent agreement is also achieved, which indicates that using the *k*-*ω* SST model could obtain reasonable and accurate results.

#### Effect of defect length on flow around airfoil

**Fig 7:**
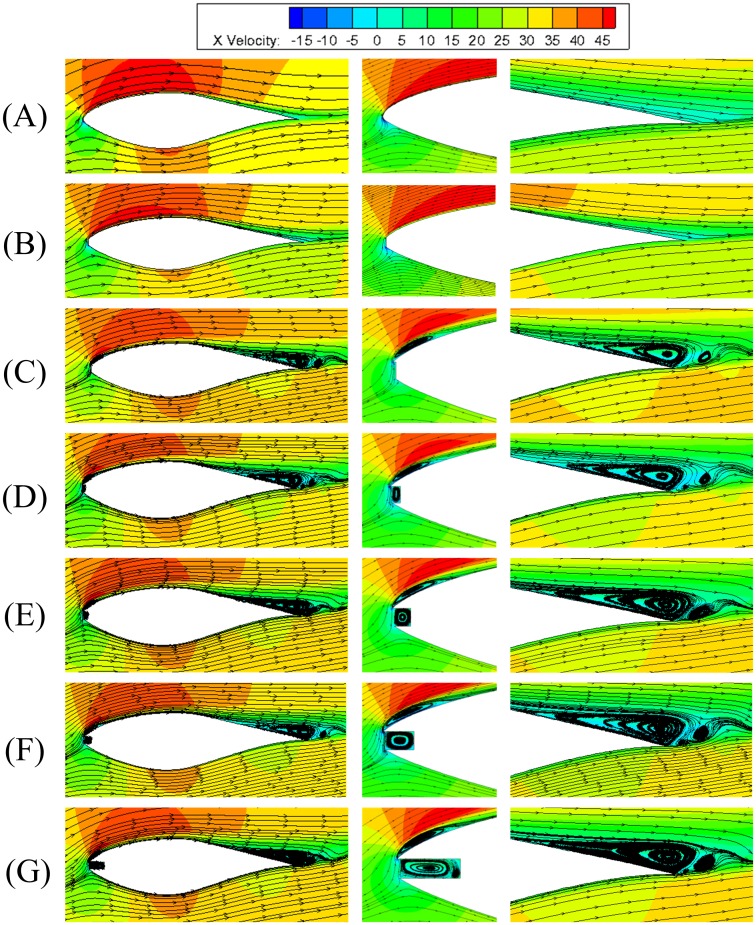
Streamlines and velocity magnitude contours for S809 airfoils with various defect lengths at the same defect thickness of *t*/*t*_c_ = 12%. (A) smooth airfoil; (B) *h/c* = 0.5%; (C) *h/c* = 1%; (D) *h/c* = 2%; (E) *h*/*c* = 3%; (F) *h*/*c* = 8%; (G) *h*/*c* = 14%.

Fig 7 presents the flow contours near the leading edge, trailing edge and the whole airfoils with various leading edge defect lengths at the same defect thickness of *t*/*t*_c_ = 12%. It can be seen that the flow is fully attached on the airfoil when *h*/*c* = 0.5% and the velocity contours is similar to that of the smooth airfoil. When the defect length is greater than or equal to 1%*c*, the flow fields for all defect airfoils are similar, i.e., a small region of separated flow can be observed near the leading edge of airfoil, which reattaches to the airfoil surface in downstream, forming a spindly separation bubble with a length of around 8%*c* near the leading edge of the suction surface. At the same time, there exists a flow separation region at 1/3 of the rear surface, forming the tailing edge vortex. The size of the leading edge separation bubble grows slowly with the increase of defect length. The tailing edge separation areas are basically the same for airfoils with a defect length greater than 1%*c*, for the appearance of the sharp edge at the upper side of the leading edge. The mainstream flow reaches the stagnation point and moves around the leading edge, where the flow detours the sharp edge and causes flow separation at the suction surface and forms the separation bubble near the leading edge. A cavity will appear when the defect extent increases, and the fluid moves over the leading edge and fills into the cavity with circulation in it. The separation bubble induced by the sharp edge changes the effective configuration and finally affects the aerodynamic performance of the airfoil.

**Fig 8:**
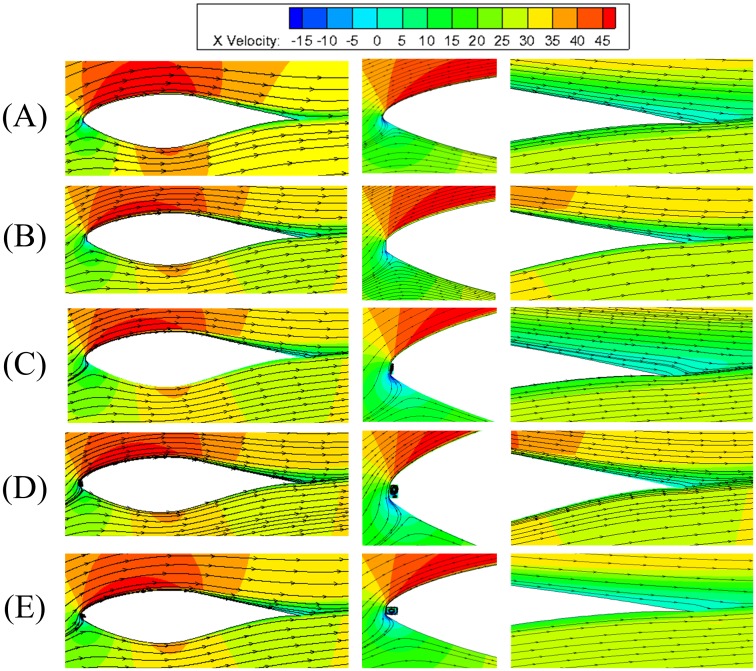
Streamlines and velocity contours for S809 airfoils with various defect lengths at the defect thickness of *t*/*t*_c_ = 6%. (A) smooth airfoil. (B) *h/c* = 0.3%. (C) *h/c* = 0.5%. (D) *h/c* = 1%. (E) *h*/*c* = 2%.

Fig 8 presents the flow streamlines and velocity contours around airfoil with various leading edge defect lengths and defect thickness of *t*/*t*_c_ = 6% at the angle of attack 8°. Attached flows around the airfoil are observed for all cases, and there are no flow separations at the leading and tailing edges, which is similar to flow around a smooth airfoil without defect. A circulation bubble can be observed in the defect cavity with length no more less than 0.5%*c*, which is 2%*c* and 3%*c* for airfoil with defect thickness of 18%*t*_c_ and 25%*t*_c_, respectively. In addition, the critical defect length for the emergence of circulation bubble is not influenced when the angle of attack is changed to 4.1°or 12.2°.

#### Effect of defect length on pressure coefficient of airfoil

**Fig 9:**
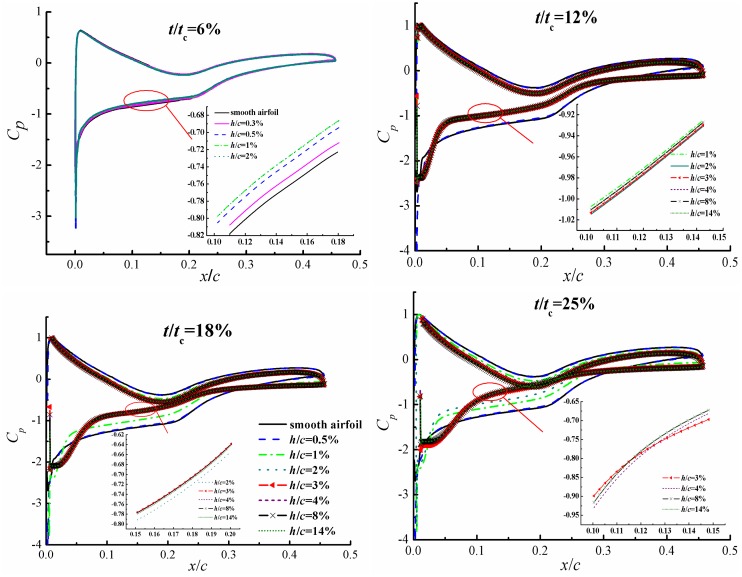
Pressure coefficient distributions of S809 airfoil with defect thicknesses of *t*/*t*_c_ = 6%, 12%, 18% and 25%.

Fig 9 presents the pressure coefficients along the airfoil with various defect lengths at the defect thicknesses of 6%*t*_c_, 12%*t*_c_, 18%*t*_c_ and 25%*t*_c_. It can be seen that the pressure coefficients are close to that of smooth airfoil for airfoils with defect thickness of 6%*t*_c_, especially when the defect length is smaller than 0.3%*c* since the flow field is little affected. For the airfoil with the defect thickness of 12%*t*_c_, significant deviations of pressure coefficients with the smooth airfoil are obtained for airfoils with defect lengths larger than 0.5%*c*. Also, the pressure distribution does not change a lot when the defect length is larger than 1%*c* with the defect thickness of 12%*t*_c_, which is in accordance with the results of flow structures in Fig 7. Similarly, the distributions of pressure coefficient are almost the same when the defect lengths are not less than 2%*c* or 3%*c* for airfoils with the defect thicknesses of 18%*t*_c_ or 25%*t*_c_, respectively. In other words, the effect of defect length on the aerodynamic characteristics of the airfoil is negligible if it reaches a critical value, which is larger for larger defect thickness.

#### Effect of defect length on the aerodynamics of airfoil

**Fig 10:**
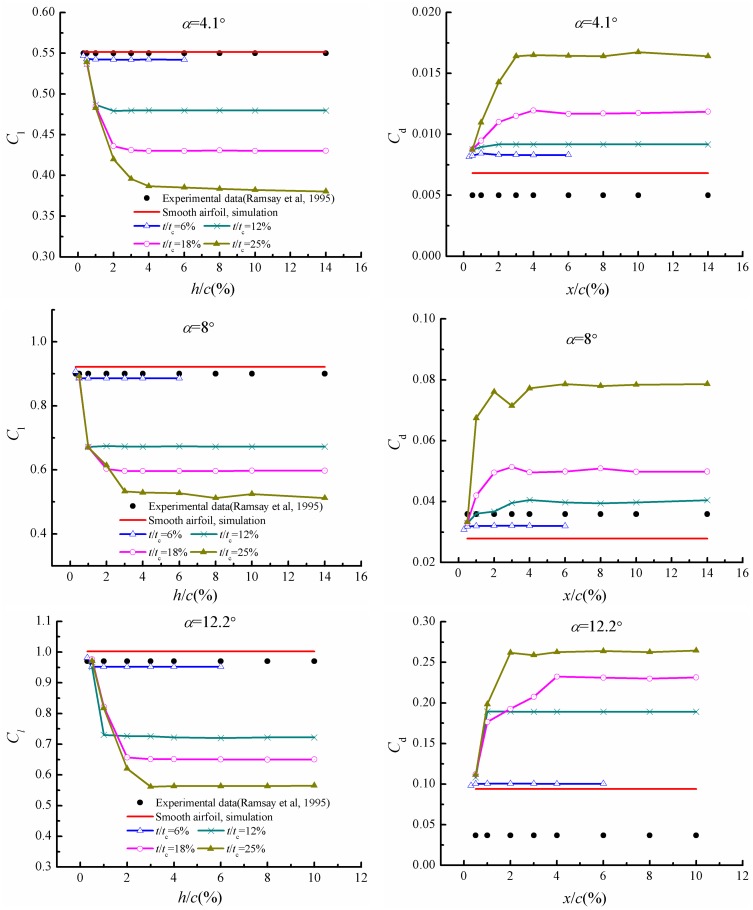
Variations of lift and drag coefficients with respect to defect length and thickness.

Fig 10 displays the variations of lift and drag coefficients for airfoils with various defect lengths and thicknesses at the angles of attack 4.1°, 8°and 12.2°. For the defect thickness of *t*/*t*_c_ = 6%, the aerodynamic coefficients are almost the same for all of 5 defect lengths, so the condition of defect lengths larger than 6%*c* is not computed. In Fig 10, it can be seen that the lift coefficients decrease while drag coefficients increase apparently at small defect lengths due to the local shape change and induced flow separation. The aerodynamic coefficients could achieve constants at the defect length larger than 0.5%*c*, 1%*c*, 2%*c*, and 3%*c* for defect thicknesses of *t*/*t*_c_ = 12%, 18% and 25%, respectively. Similar trends at three angles of attack of 4.1°, 8°and 12.2° are observed. At the angle of attack of 4.1°, the lift coefficient decreases about 0.87% with defect length of *h*/*c* = 0.3% and approximately 1.7% if the defect length is greater than 0.5%*c* at the defect thickness of *t*/*t*_c_ = 6%. For the defect thickness of *t*/*t*_c_ = 12%, the lift coefficients decrease about 2.6% at the defect length of 0.5%*c* and approximately 13% when the defect length is greater than 1%*c*. For the defect thickness of 18%*t*_*c*_ and 25%*t*_*c*_, the lift decreases are close to each other with defect lengths of 0.5%*c* and 1%*c*, and the lift coefficients drop about 2.7% and 12.1%, respectively. The lift decreases about 21% and 24% for airfoil with defect thicknesses of 18%*t*_c_ and 25%*t*_c_ at the defect lengths of 2%*c*, and the lift decrease will be up to 22% and 30% when the defect lengths are greater than 2%*c* and 3%*c*. At the angle of attack 8°, the lift decreases about 1.5% for airfoil with the defect thickness of 6%*t*_c_ at the defect lengths of 0.3%*c* and will reach to 3.6% when the defect length is greater than 0.5%*c*. The lift decrease about 26%, 35% and 42%, while the drag coefficient increases about 42%, 85% and 157% for airfoils with a defect length greater than 1%*c*, 2%*c* and 3%*c* at the defect thicknesses of 12%*t*_c_, 18%*t*_c_ and 25%*t*_c_, respectively. The lift and drag coefficients demonstrate similar variations at the angles of attack 12.2°, 4.1°and 8°. The lift decrease will reach up to 5%, 28%, 35% and 44% when the lift coefficients remain constants for airfoils with the defect lengths of 6%*t*_c_, 12%*t*_c_, 18%*t*_c_ and 25%*t*_c_, respectively, while the drag coefficients increase about 4.5%, 102%, 148% and 178% at the same conditions. The defect thickness of 6%*t*_c_ has a relative small influence on the aerodynamics of S809 airfoil.

By comparing the lift and drag coefficients for airfoils with the same defect size at the three investigated angles of attack, it can be perceived that the influence of defect grows with the increase angle of attack. For the airfoil with the defect length of 3%*c* and defect thickness of 12%*t*_c_, the lift coefficients decrease about 13%, 26% and 28% at the angles of attack 4.1°, 8°and 12.2°, while the drag coefficients increase about 35%, 42% and 102%, respectively. The results indicate that leading edge defect has a significant influence on the aerodynamic coefficients of airfoil, which can remain constant when the defect length reaches to a critical value. Meanwhile, the effect of defect grows with the increase of angles of attack.

#### Critical influence defect length/thickness

**Fig 11:**
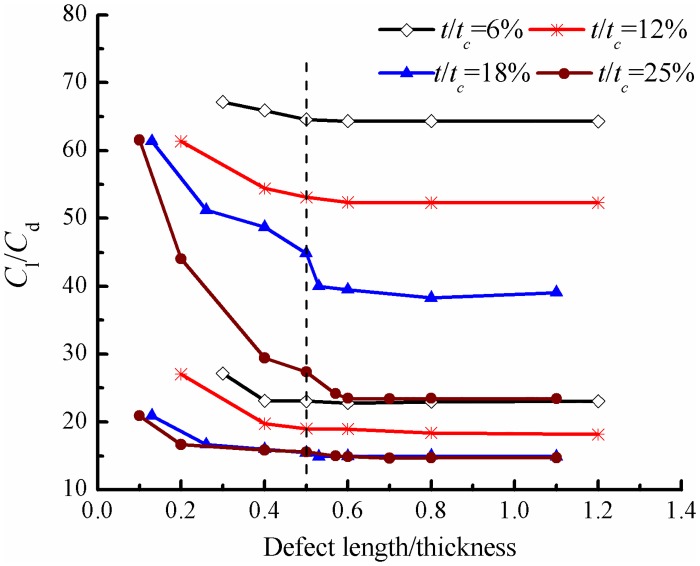
Lift/drag coefficients for airfoils with various defect length/thickness.

In order to determine the critical aspect ratio between defect length and thickness at which the effect of leading edge defect on the aerodynamics of the airfoil does not change anymore, a series of defect aspect ratios ranging from 0.1 to 1.2 are considered in the computation under different defect thicknesses and angles of attack, and the results are shown in Fig 11. It can be seen that although the *C*_l_/*C*_d_ varies in a wide range from about 10 to 70, it remains a constant when the defect length/thickness ratio is larger than 0.5. Therefore, it can be concluded that the defect length/thickness ratio of 0.5 is the critical value for this kind of leading edge defect, above of which the aerodynamics will not change anymore.

### Dynamic case

#### Numerical validation

**Fig 12:**
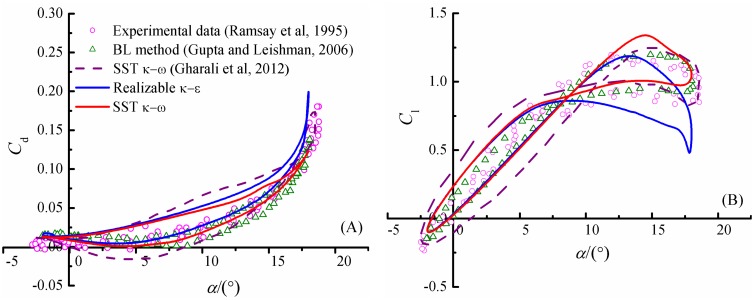
Drag and lift coefficients for smooth airfoil in dynamic stall case. (A) drag coefficients. (B) lift coefficients.

The unsteady cases are computed with *α*_mean_ = 8° and *α*_amp_ = 10° in order to make direct comparison with previous studies. The computed drag and lift coefficients by the *k*-*ω* SST and realizable *k*-*ε* models are displayed in Fig 12, as well as experimental data result of Ramsay et al[23], computational results[3, 25]. The present predictions of the hysteretic lift-drag curves by the two models coincide generally with existed published data. The drag curve is a little over predicted at upstroke, while excellent agreement can be achieved at downstroke especially for results obtained by the *k*-*ω* SST model.

#### The unsteady flow fields

The unsteady flow in dynamic stall is a very complex fluid dynamic phenomenon. Attached flow at low angle of attack, unsteady laminar/turbulent boundary layer transition, massive flow separation, vortex development and its shedding, as well as flow reattachment make the detailed understanding of this physical flow process very difficult[14]. Due to the general process of unsteady flow development during dynamic stall of an airfoil with leading edge defect is little different from that of a smooth airfoil, the whole process of flow characteristics is not given here, and demonstrations are given only at three typical angles of attack.

**Fig 13:**
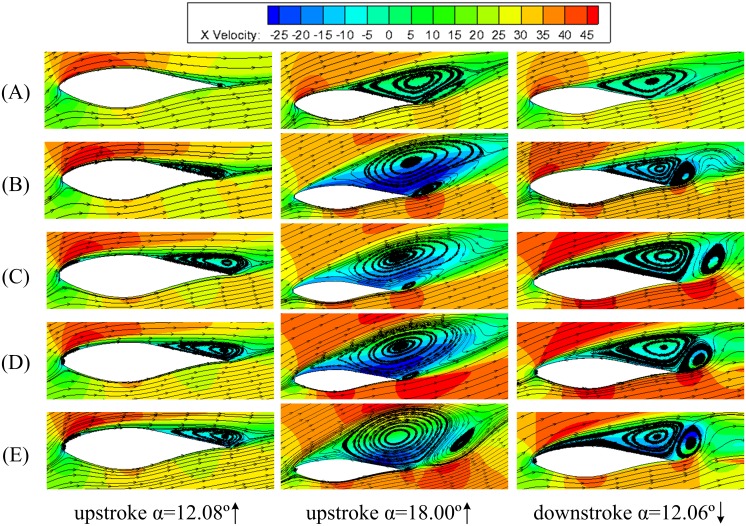
Typical flow field contours of airfoils with various defect lengths at the defect thickness of *t*/*t*_c_ = 12%. (A) smooth airfoil. (B) *h/c* = 0.5%. (C) *h/c* = 1%. (D) *h/c* = 2%. (E) *h*/*c* = 3%.

Fig 13 shows the velocity magnitude contours and streamlines for S809 airfoil with various defect lengths at upstroke angle of 12.08°, maximum angle of *α* = 18° and down-stroke angle of 12.06° with the defect thickness of 12%*t*_c_. Here *α* = 12.08° is the time when flow separation is firstly observed for smooth airfoil. The tailing edge separation region is spanned over 1/3 of the upper suction surface for airfoil with leading edge defect. The size of the tailing edge separation region with defect length greater than 1%*c* is almost the same at *α* = 12.08° during upstroke, and which is obviously larger than that of airfoil with only 0.5%*c* defect length. When the angle of attack reaches peak angle *α* = 18°, the airfoil enters into deep stall and secondary vortex is induced at the tailing edge. It can be seen that the separation region covers the whole suction surface for airfoil with leading edge defect, which just spans 1/2 of the upper surface for the smooth airfoil. In addition, with the increase of defect length, the size of separation region becomes larger, which indicates that leading edge defect may impose strong disturbance on downstream flow and cause the separation location moving forward. For *α* = 12.06° at downstroke, the flow field is more complicated, where the vortex shedding exists at the suction surface. The flow characteristics of airfoils with the defect length greater than 1%*c* are similar.

The key difference of dynamic stall process for airfoils with various defect lengths is that when the defect length is greater than 1%*c*, there will be a spindly separation region emerged near the leading edge, which cannot be detected in the flow around the smooth airfoil and small defective airfoil, as in Fig 13. Moreover, the time for the appearance of flow separation at leading edge, trailing edge separation and secondary vortex are different for airfoils with different defect length. The separation bubble at the leading edge can be detected at the angles of attack of about 6.69°, 6.41° and 6.07°, meanwhile, the tailing edge separation emerges at angles of attack of approximately 12.08°, 11.57°, 10.00°, 9.77° and 9.09° for smooth, 0.5%*c*, 1%*c*, 2%*c* and 3%*c* defective airfoils at upstroke respectively. The disappearances of the tailing edge separation are at angles of attack 6.75°, 5.35°, 4.58°, 4.26° and 3.73° at downstroke phase for airfoils with increasing defect levels. This indicates that the defect and sharp edge at the leading edge appear to act as a main driving force which induces earlier flow separation and secondary vortex shedding.

#### The aerodynamic characteristics

**Fig 14:**
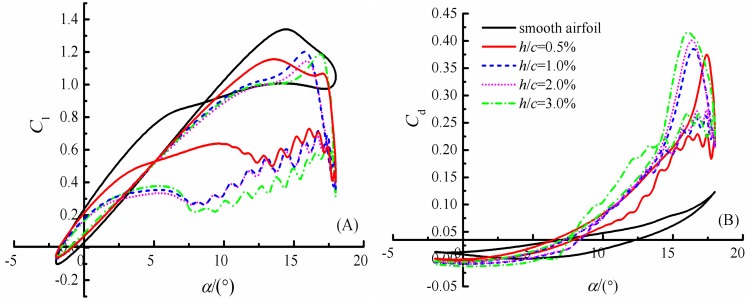
Lift and drag coefficients for airfoil with various defect lengths over S809 airfoil. (A) lift coefficients. (B) drag coefficients.

Fig 14 presents the lift and drag coefficients for S809 airfoil with various defect lengths at the defect thickness of *t*/*t*_c_ = 12%. When the defect length increases, the lift coefficient decreases. The lift curves overlap with each other until the angle of attack reaches approximately 7° at upstroke, where the lift coefficients begin to detach from each other. In other words, the effect of leading edge defect on lift is very small when the angles of attack smaller than 7° during upstroke. Meanwhile, airfoils with defect length greater than or equal to 1%*c* present similar trends for lift coefficient hysteresis loops, which is a little difference from that of airfoil with 0.5%*c* defect length or smooth. The lift exhibit a sharp drop-off shortly after the peak value at the angles of attack around 15.78°, 15.92° and 16.85° for airfoils with defect lengths of 1%*c*, 2%*c* and 3%*c*, respectively. This sharp decrease may due to the generation, shedding of the secondary vortex and couple with the rotating of the main vortex at the suction surface during dynamic process. All curves show slight fluctuations at high angles of attack during downstroke phase, this phenomenon maybe attributed to the shedding of the secondary vortices.

The drag coefficients increase dramatically for airfoils with leading edge defect when compared to the smooth airfoil, as in Fig 14. During the upstroke, the drag curves for all airfoils are almost the same until the angle of attack reaches approximately 7°, where the drag coefficients increase sharply for defective airfoils and meet the peak value at around 16° angle of attack. With the increase of defect length, the drag coefficients also increase, and the average drag increase about 118.38%, 126.04%, 132.37% and 157.01%, while the lift reductions are 23.95%, 34.46%, 34.93%, and 36.11% for airfoils with defect length of 0.5%*c*, 1%*c*, 2%*c* and 3%*c*, respectively.

## Conclusions

In this paper, we have presented a numerical study of defect effects on aerodynamics and flow characteristics of an S809 airfoil both in both static and dynamic conditions at a Reynolds number of 1×10^6^. The computation is conducted with the *k*-*ω* SST turbulence model, and validated by comparison with previous experimental and computational data of normal airfoil without defect [3, 23, 25]. The influence of leading edge defects on flow characteristics, pressure coefficients, aerodynamics are investigated, and a critical defect length/thickness ratio is obtained. More defect size parameters close to actual conditions are considered in comparison with previous studies. The present computational results demonstrate that the aerodynamic coefficients and the flow characteristics are sensitive to leading edge defect.

In steady state case, the lift coefficient decreases while drag coefficient increases of defective airfoil compared with the normal one except for the defect thickness of 6%*t*_c_, in which a relatively small influence of leading edge defect has been observed. For other defect thicknesses, the existence of defect leads to the emergence of a circulation bubble in the defect cavity and trailing edge separation, while the flow characteristics could keep unchanged when the defect length reaches certain value. The pressure distribution and aerodynamic coefficients also do not change a lot when the defect lengths are not less than 0.5%*c*, 1%*c*, 2%*c* and 3%*c* for airfoils with defect thicknesses of 6%*t*_c_, 12%*t*_c_, 18%*t*_c_ and 25%*t*_c_, respectively. The critical defect length/thickness ratio is 0.5 for all defect sizes and beyond which the defect influence keeps unchanged, thus it can be considered as a universe parameter to characterize the effect of this kind of defect.

In the dynamic stall conditions, the influence of leading edge defect demonstrates significant influence on the aerodynamic coefficients when the angle of attack is larger than 7° during upstroke. The peak angle of attack grows with the increase of defect length, which is in accordance with flow separation spanning over the whole suction surface. The lift reductions are approximately 24%, 34%, 35%, and 36% for airfoils with defect lengths of 0.5%*c*, 1%*c*, 2%*c* and 3%*c*, respectively, which is basically similar to the results in steady state case, while the drag increase is much greater than the steady condition.

Since the profile of the defective wind turbine blade is very complex in real cases, the authors just employed a much simplified geometry in current study to investigate the influence of leading edge defect on the aerodynamic characteristics of an airfoil. Studies on other various defect or erosion models which are much closer to real conditions or three-dimension eroded blades are the critical issues to consider in future.

## Supporting Information

S1 File Supporting information legends. The file list of the supporting materials.
(DOCX)

S2 File Dataset. The file contains the procedure adopted for calculating the velocity inlet at each time step used in Fig 4, and data adopted to draw the Figs 5, 6, 9–12 and 14.
(RAR)
