# Supplementary material for: Effects of Leading Edge Defect on the Aerodynamic and Flow Characteristics of an S809 Airfoil
Source: PLoS One. 2016 Sep 22;11(9):e0163443. doi: 10.1371/journal.pone.0163443 (PMC5033410; doi:10.1371/journal.pone.0163443)
Supplement: S1 File — The file list of the supporting materials. (DOCX) [file pone.0163443.s001.docx]

**Supporting information files**

**S1 Fig. Sinusoidal inlet velocity of the computational domain**

The figure shows the inlet velocity at each time step used in the simulation, including the horizontal velocity and vertical velocity. The procedure using for calculating the illustration of velocity inlet is attached in the supporting materials, which is compiled with FORTRAN and provided as the format of .txt (TXT).

**S2 Fig. Lift and drag coefficients versus *α* for a smooth airfoil in steady case.** **(A) lift coefficient. (B) drag coefficient.**

The figure shows the comparison of lift and drag coefficients versus *α* for the S809 airfoil without defect. (A) lift coefficient. (B) drag coefficient. The data can be obtained from ‘Raw data of Fig 5’ (DOC).

**S3 Fig. Pressure coefficient computed by *k*-*ω* SST turbulence model**

The figure shows the pressure coefficient computed by k-ω SST turbulence model and data obtained from the wind tunnel experiment at the angles of attack 8.1〫 and 15〫. The data can be obtained from ‘Raw data of Fig 6’, (XLSX).

**S4 Fig. Pressure coefficient distributions of S809 airfoil with defect thicknesses of *t*/*t*_c_=6%, 12%, 18% and 25%.**

The figure shows the pressure coefficients of airfoils with various defect lengths at the defect thicknesses of *t*/*t*_c_=6%, 12%, 18% and 25%, respectively, where t is the defect thickness of the airfoil, and ***t*_c_** is the thickness of airfoil. The data can be obtained from the ‘Raw data of Fig 9’ (XLSX).

**S5 Fig. Variations of lift and drag coefficients with respect to defect length and thickness**

The figure shows the lift and drag coefficients variations with respect to defect lengths for airfoils with various defect thickness at the angles of attack 4.1〫, 8〫 and 12.2〫.

The data can be obtained from ‘Raw data of Fig 10’, (DOC).

**S6 Fig. Lift/drag coefficients for airfoils with various defect length/thickness**

The figure shows the value of lift/drag coefficients of airfoil with various defect ratio of length/thickness, the data can be obtained from ‘Raw data of Fig 11’, (XLSX).

**S7 Fig. Drag and lift coefficients for smooth airfoil in dynamic stall case. (A) drag coefficients. (B) lift coefficients.**

The figure shows the comparison of drag and lift coefficients obtained from calculation and experimental data. The data can be obtained from ‘Raw data of Fig 12’, (XLSX).

**S8 Fig. Lift and drag coefficients for airfoil with various defect lengths over S809 airfoil. (A) lift coefficients. (B) drag coefficients.**

The figure shows the lift and drag coefficients for airfoil with various defect lengths and the defect thickness of *t*/*t*_c_=12% at dynamic stall conditions. The data can be obtained from ‘Raw data of Fig 14’, (XLSX).
